# Negative Pressure Wound Therapy with Instillation and Dwell for the Management of a Complex Burn: A Case Report and Review of the Literature

**DOI:** 10.7759/cureus.3514

**Published:** 2018-10-29

**Authors:** Pablo L Padilla, Elliott P Freudenburg, Katarzyna Kania, Rece W Laney, Ludwik K Branski, David N Herndon

**Affiliations:** 1 Plastic and Reconstructive Surgery, The University of Texas Medical Branch, Galveston, USA; 2 Miscellaneous, The University of Texas Medical Branch, Galveston, USA; 3 Plastic and Reconstructive Surgery, Baylor College of Medicine, Houston, USA

**Keywords:** wound vac, instillation vac, burn care, veraflo, wound vac burns, whole body vac, complex wound, wound care

## Abstract

Research over the last 50 years has led to significant improvements in outcomes for burn victims. Advances in infection control, attenuation of the hypermetabolic response, and new improved surgical approaches have led to decreased morbidity and mortality. Early wound excision eliminates the devitalized tissue, which is the main reservoir for pathogen propagation. Immediate autografting reestablishes the natural barrier of the skin, which blocks pathogen access to the host. Advances in burn care have increased treatment options for patients with devastating injuries presenting with multiple comorbidities. Over the last 20 years, negative pressure assisted wound therapy (NPWT) has shown to improve wound management and healing as well as decrease the length of recovery in burn patients. As NPWT applications evolve, the development of negative pressure wound therapy with instillation and dwell time (NPWTi-d) for the management of complex and infected wounds has proven vital for patient care.

We present the case of a 68-year-old male patient presenting with a three-day-old third-degree burn wound spanning 46% of the total body surface area (TBSA). After the infected wound was treated unsuccessfully with the standard of care (excision, debridement, and grafting), the team utilized NPWTi-d in order to mitigate the infection and promote the formation of granulation tissue, leading to the successful grafting of the burn wound.

NPWTi-d was a useful adjunct in treating and stimulating wound healing in a complex patient. This is the first case report of its kind, utilizing a whole-body vacuum assisted closure (VAC) with NPWTi-d, with successful results showing a decreased bacterial burden, decreased morbidity and mortality, and patient wound closure.

## Introduction

Burns are one of the most common and devastating forms of trauma [1-2]. Burns remain one of the most prevalent and preventable public health problems globally. In North America, burn injuries remain a major cause of death with the highest fatality rate seen in the elderly and children under the age of four [3-4]. In 2014, a total of 3,194 deaths occurred from fire and burns alone, representing a mortality rate of 1/100,000 population or 1.6% of all injury fatalities. Immediate mortality from burn trauma is part of the problem, but patients that survive the acute trauma state tend to face serious infections and life-threatening sepsis [5].

Large thermal injuries induce a state of immunosuppression that predisposes patients to a multitude of complications and severe infections [6]. Different therapy and treatment strategies are employed worldwide for burn injuries, with the end goal being the survival of the patient.

## Case presentation

**Figure 1:**
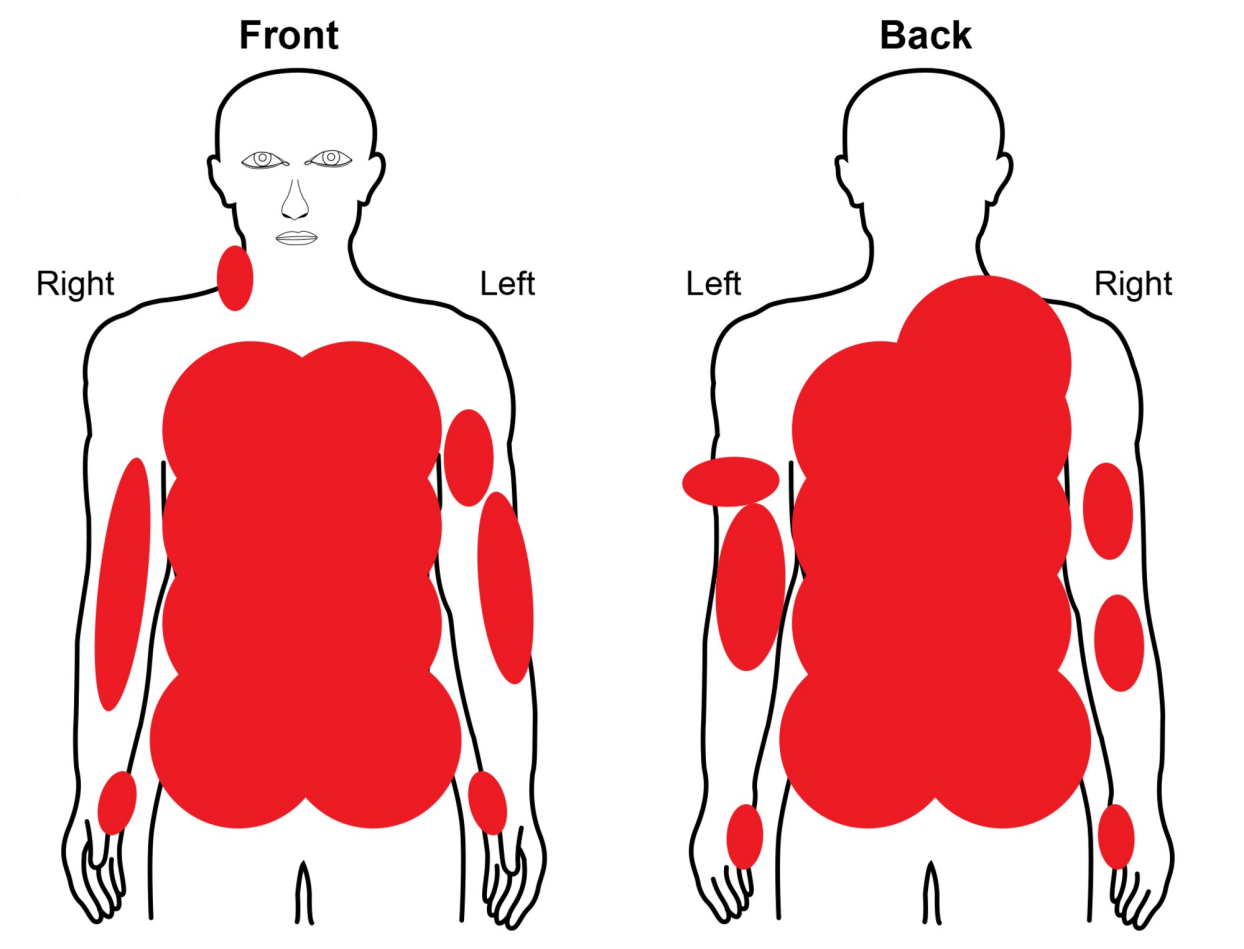
Admission Burn Diagram

A 68-year-old male presented with a three-day-old, 46% total body surface area (TBSA), third-degree burn to the flank, bilateral upper extremities, and back (Figure 1).

**Figure 2:**
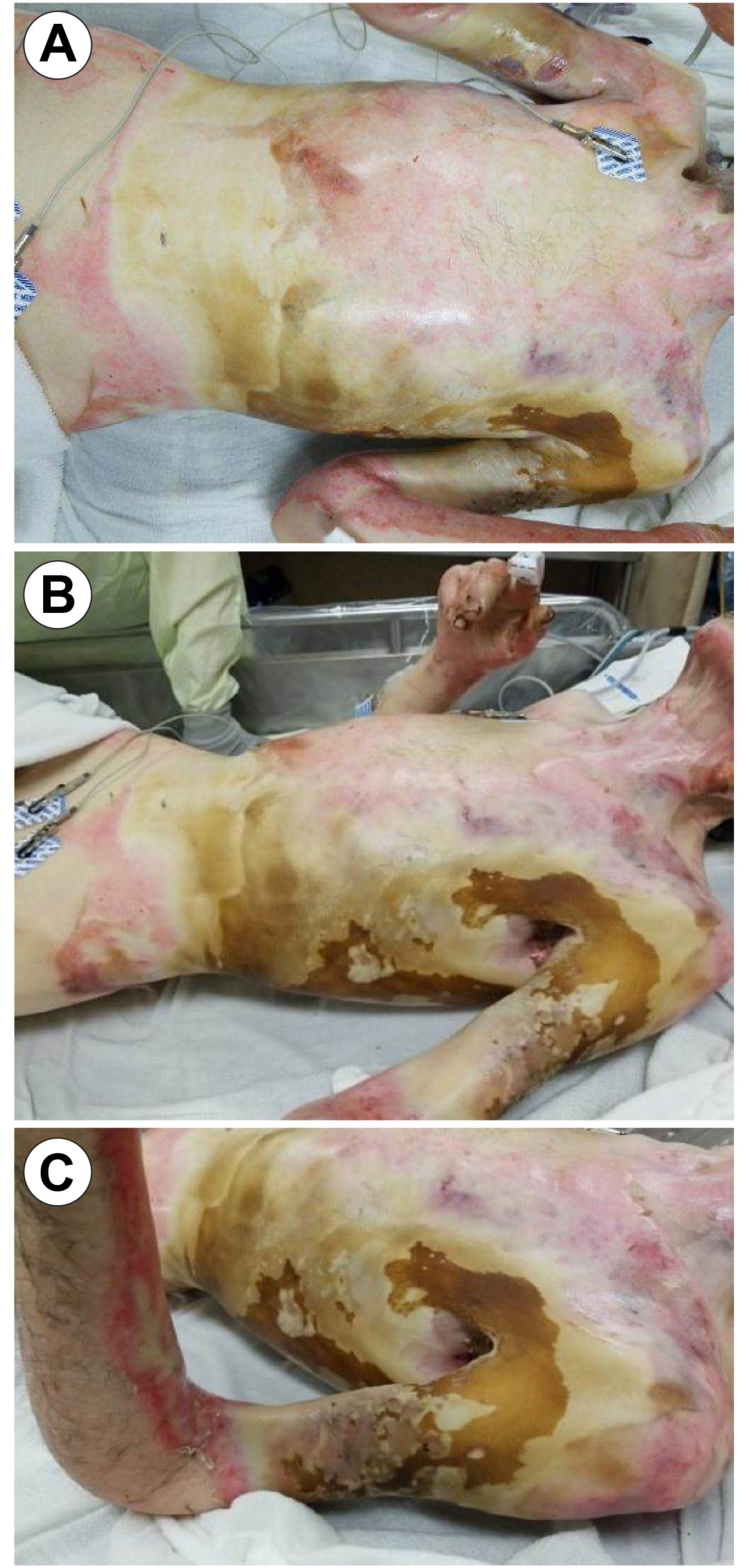
Admission Images (Post-Burn Day 3) Image A: Anterior Posterior View; Image B: View of Left Lateral Burn Eschar (Anterior Torso); Image C: View of Left Upper Extremity Burn Eschar

Past medical history consisted of hypertension and severe anorexia, with a body mass index (BMI) of 14. The patient suffered a flame burn when his clothes caught fire in a home furnace/heating apparatus. He stayed at home for two days post-injury, refusing treatment, however, on day three, he was found to be in excruciating pain with altered mental status. His partner called emergency services, and he was admitted through our hospital burn emergency department. Upon initial presentation, he was resuscitated, stabilized, and taken for emergent excision debridement and grafting of the burn eschar and affected tissue, according to institution policy (Figure 2).

**Figure 3:**
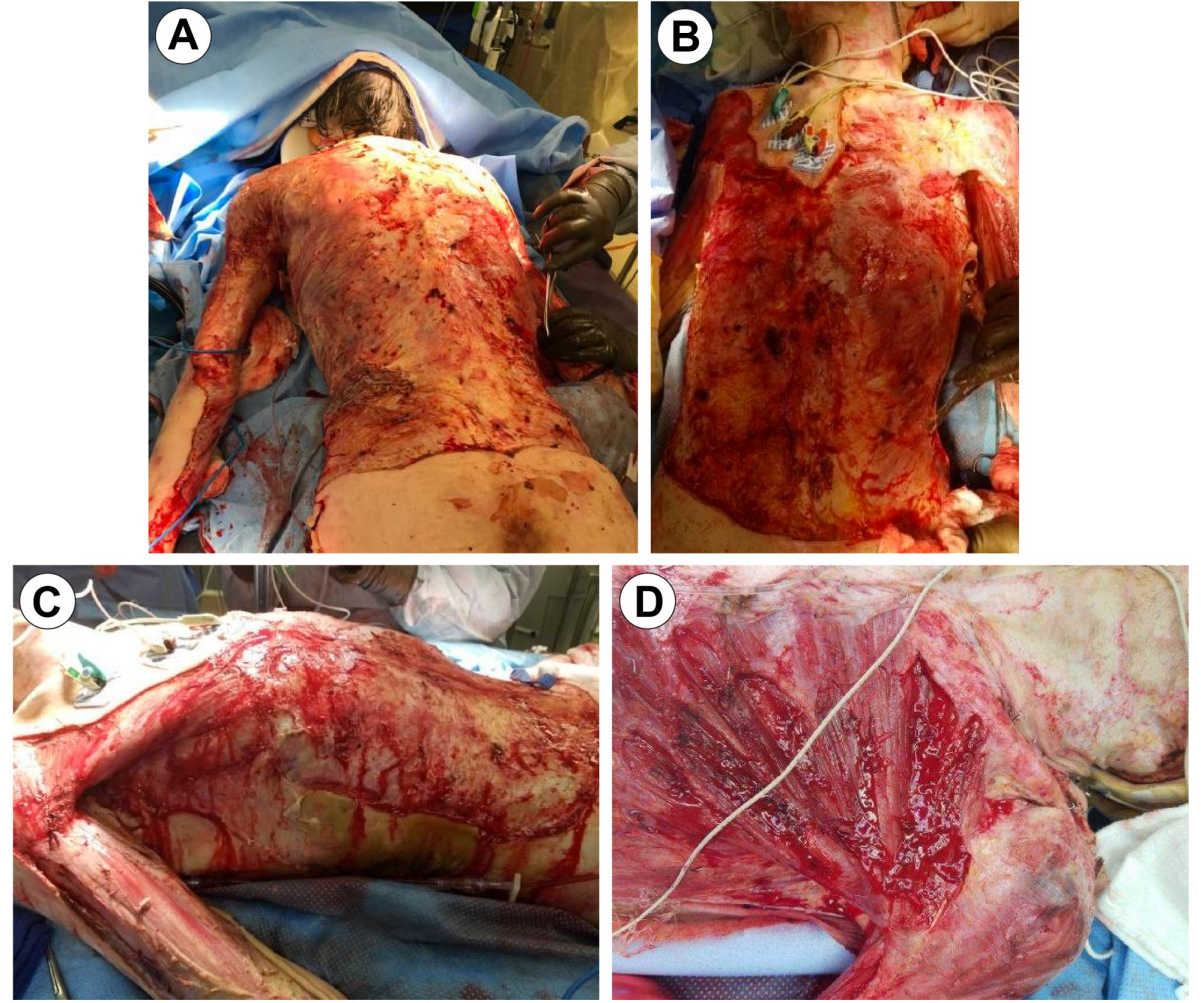
OR Debridement Day 1 Image A: Posterior Torso View; Image B: Anterior Torso View; Image C: Right Torso; Image D: Close-Up (Left Chest) Exposed Musculature/Periosteum OR: Operating Room

He was taken to the operating room on admission day 2 for excision, debridement, and grafting of all burn and affected tissue. Autograft was harvested from the scalp and bilateral lower extremities, meshed in a 4:1 and 2:1 cadaveric homograft overlay, the so-called sandwich technique [7], and applied over burned areas. The depth of the patient's wound extended to the rib periosteum, with exposed necrotic muscle on the trunk and bilateral upper extremities (Figure 3).

The standard wound care regimen, with broad-spectrum intravenous antibiotics, topical antibiotics, and soaks, was implemented. 

**Figure 4:**
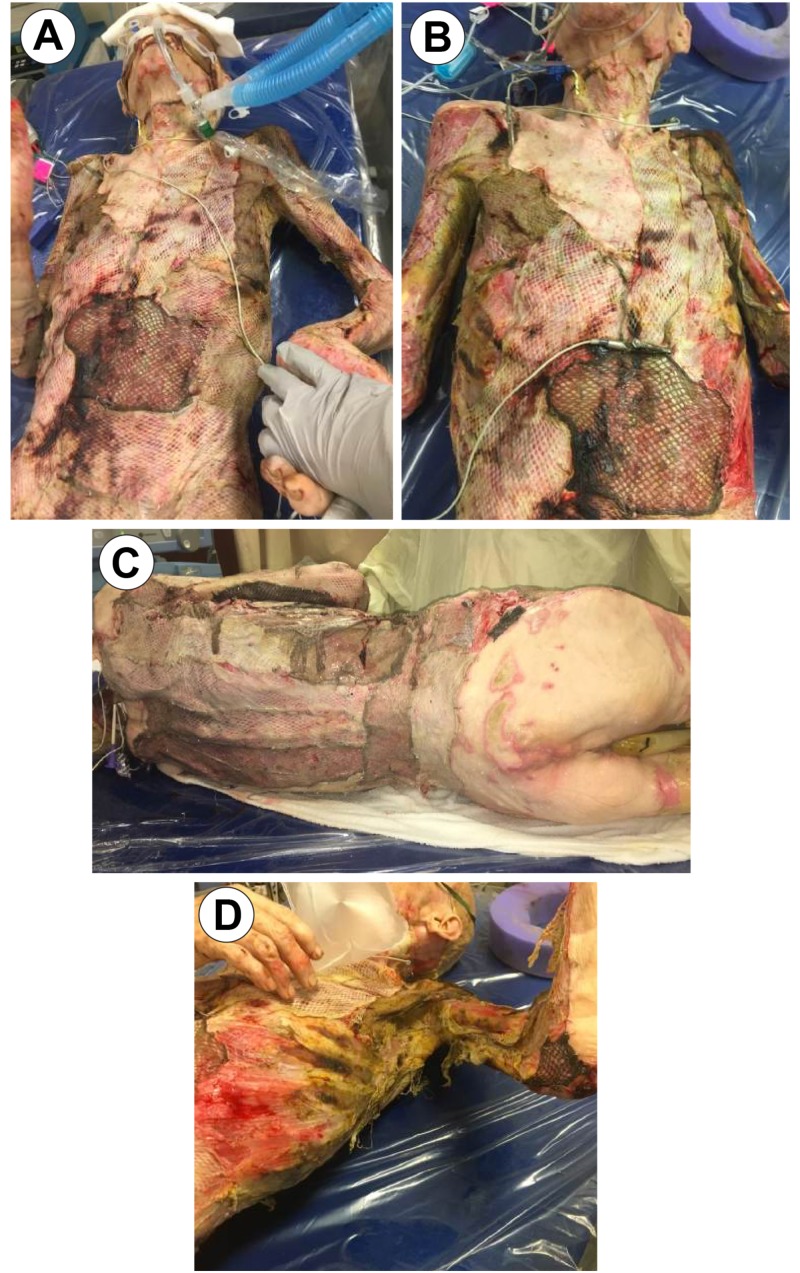
OR Day 7 Image A: Anterior Torso; Image B: Anterior Torso; Image C: Posterior Torso; Image D: Left Axillary/ Chest View OR: Operating Room

On his second trip to the operating room, one-week post-op, grafts showed an almost 95% graft failure (Figure 4) with quantitative tissue cultures growing *Escherichia  coli*, *Enterobacter*, *Acinetobacter*, and *Klebsiella*.

The patients' critical status, impaired nutritional state, and infection burden warranted alternative therapy options, thus, the team elected to try negative pressure wound therapy with dwell time (NPWTi-d) using the KCI Veraflo (KCI Medical, San Antonio, Texas, US) system. 

**Figure 5:**
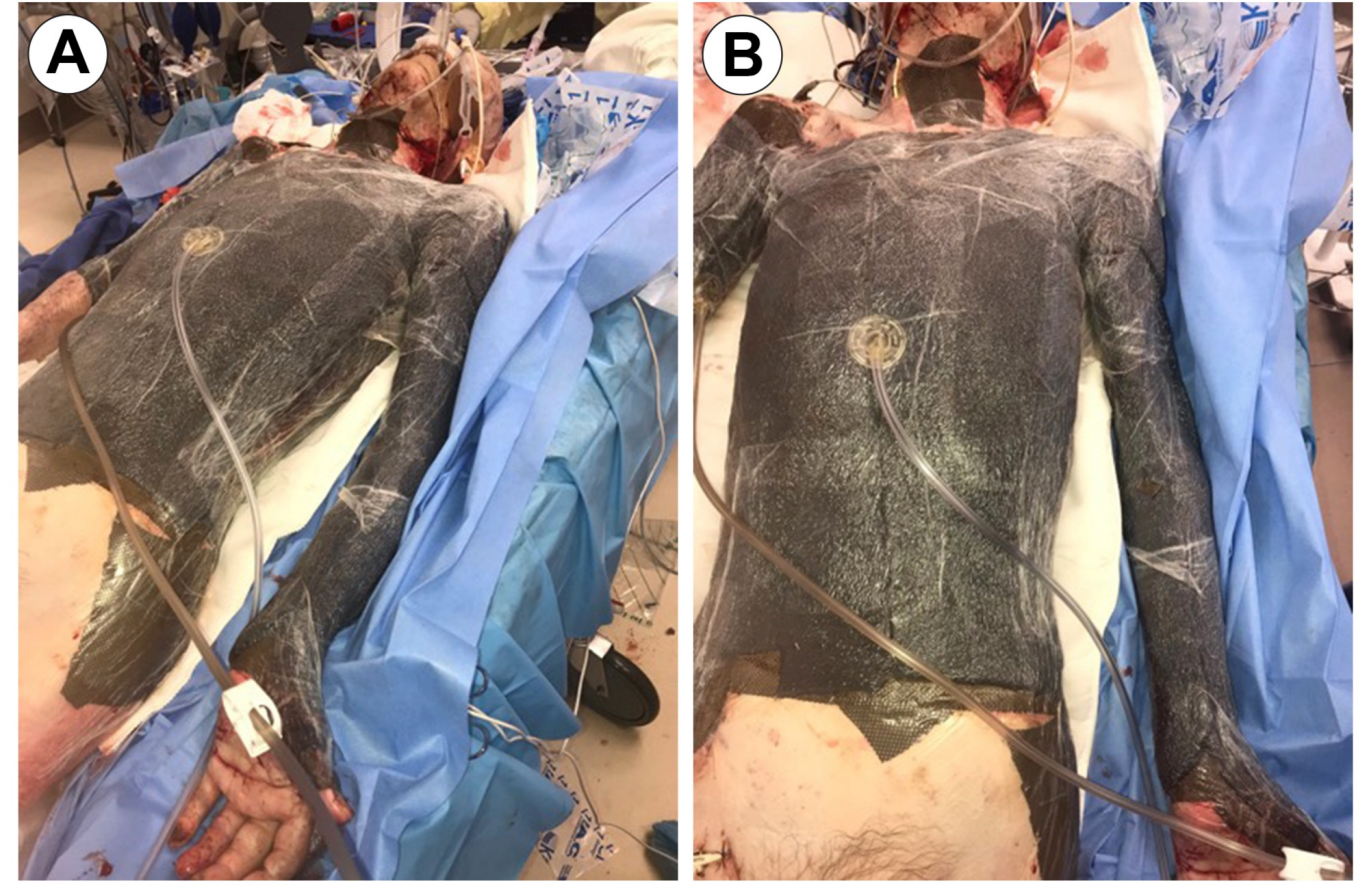
Veraflo Application Image A: Full Body VAC Placement Anterior View; Image B: Full Body VAC Placement Anterior View 2 VAC: Vacuum assisted closure

Procedure detail: Black foam sponge was shaped to fit the bilateral upper extremities and torso. Two Veraflo devices were employed: one for the bilateral upper extremities connected utilizing a “y” connector and the second for the torso. The team created two independent and separate suction entities: the bilateral upper extremities and the anterior/posterior torso (Figure 5).

Therapy settings were as follows: 18 min dwell time, every 2.5 hours, with a constant pressure of 125 mmHg, utilizing VASHE hypochlorous solution (SteadMed Medical, Fort Worth, Texas, US). Therapy settings were calculated with the assistance of KCI representatives and a review by McKanna et al. which provided information on therapy settings that were adapted to our particular patient. The large wound bed, highly exudative nature of the burn injury, and difficulty in maintaining seal integrity all contributed to the indicated therapy settings [8]. The solution utilized was due to a hospital safety protocol implemented to prevent the inadvertent administration of similar appearing solutions and medications. The indicated VASHE hypochlorous solution was provided by the hospital pharmacy in blue bottles with readymade caps for introduction into the Veraflo device for seamless therapy.

**Figure 6:**
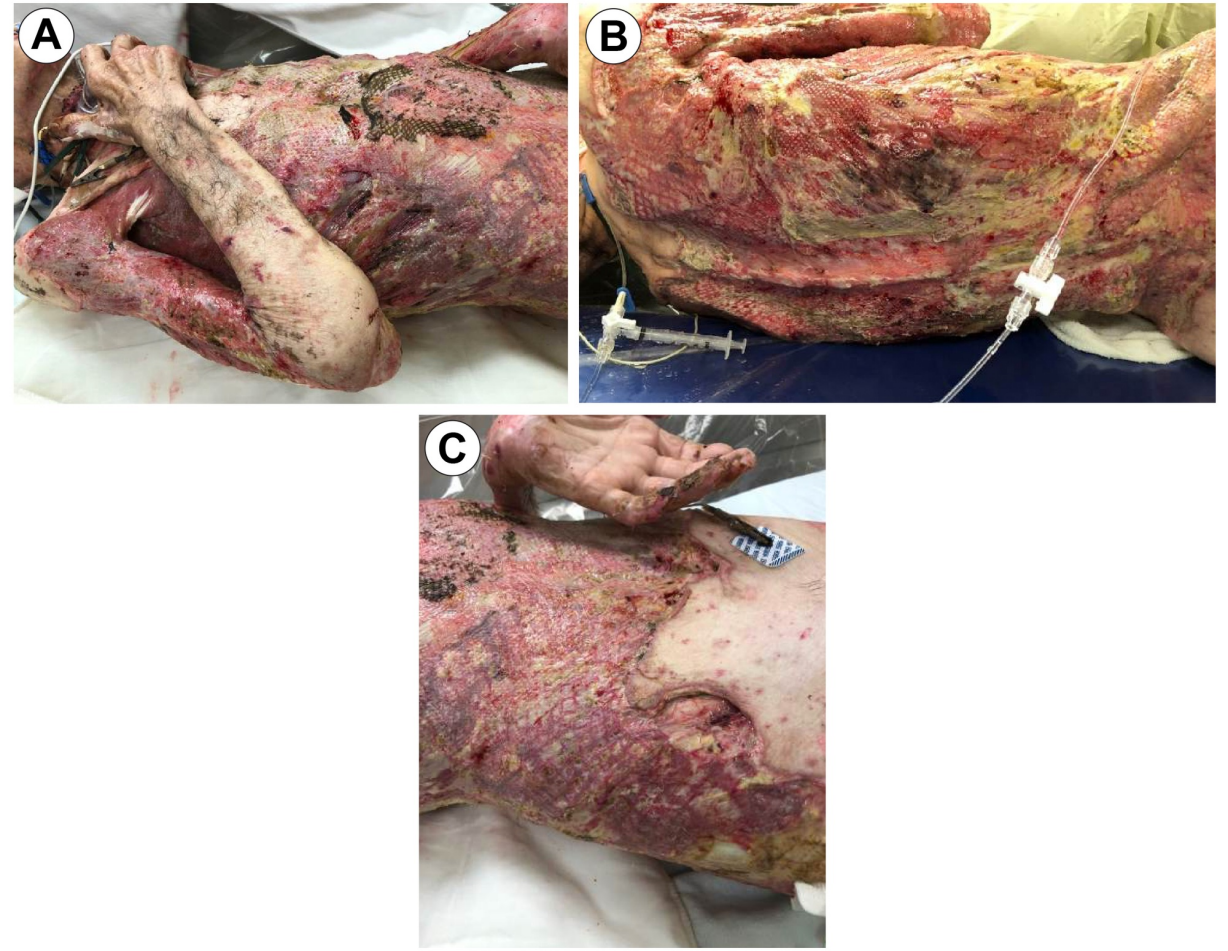
Two Weeks Post VAC Therapy Image A: Right Anterior Torso View; Image B: Posterior Torso View; Image C: Zoom of Anterior Torso VAC: Vacuum assisted closure

**Figure 7:**
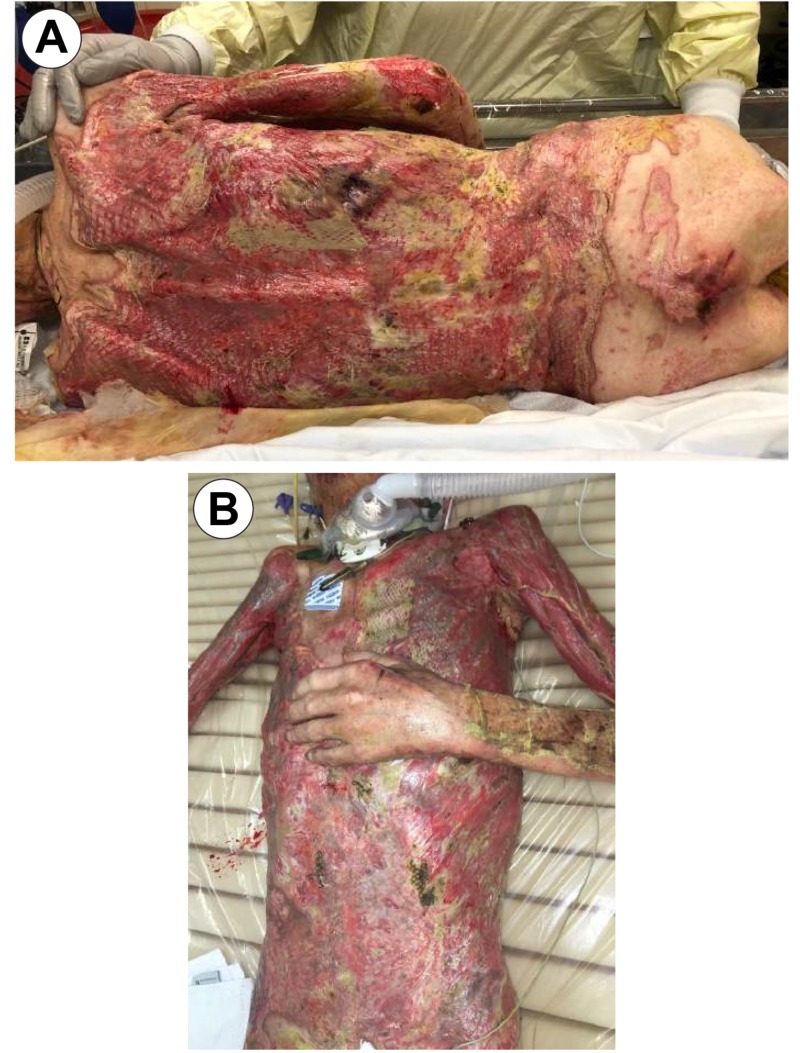
Two Weeks Post VAC Therapy Image A: Posterior Torso; Image B: Anterior Torso VAC: Vacuum assisted closure

In between operating room cases, standard burn intensive care was employed with an emphasis on patient rotation every two hours per institution policy to avoid pressure sore formation and the vacuum assisted closure (VAC) devices were checked every hour for signs of copious bleeding or malfunction (loss of suction, machine malfunction, or therapy issues). He was taken to the operating room for VAC exchange, assessment of wounds with cultures, and further debridement every 72-96 hours (Figure 6-7).

**Figure 8:**
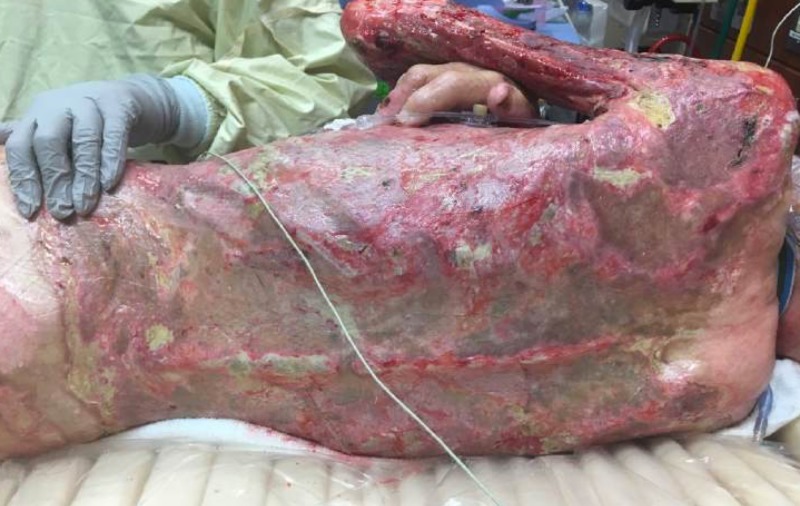
Six Weeks Post Burn Posterior Torso

**Figure 9:**
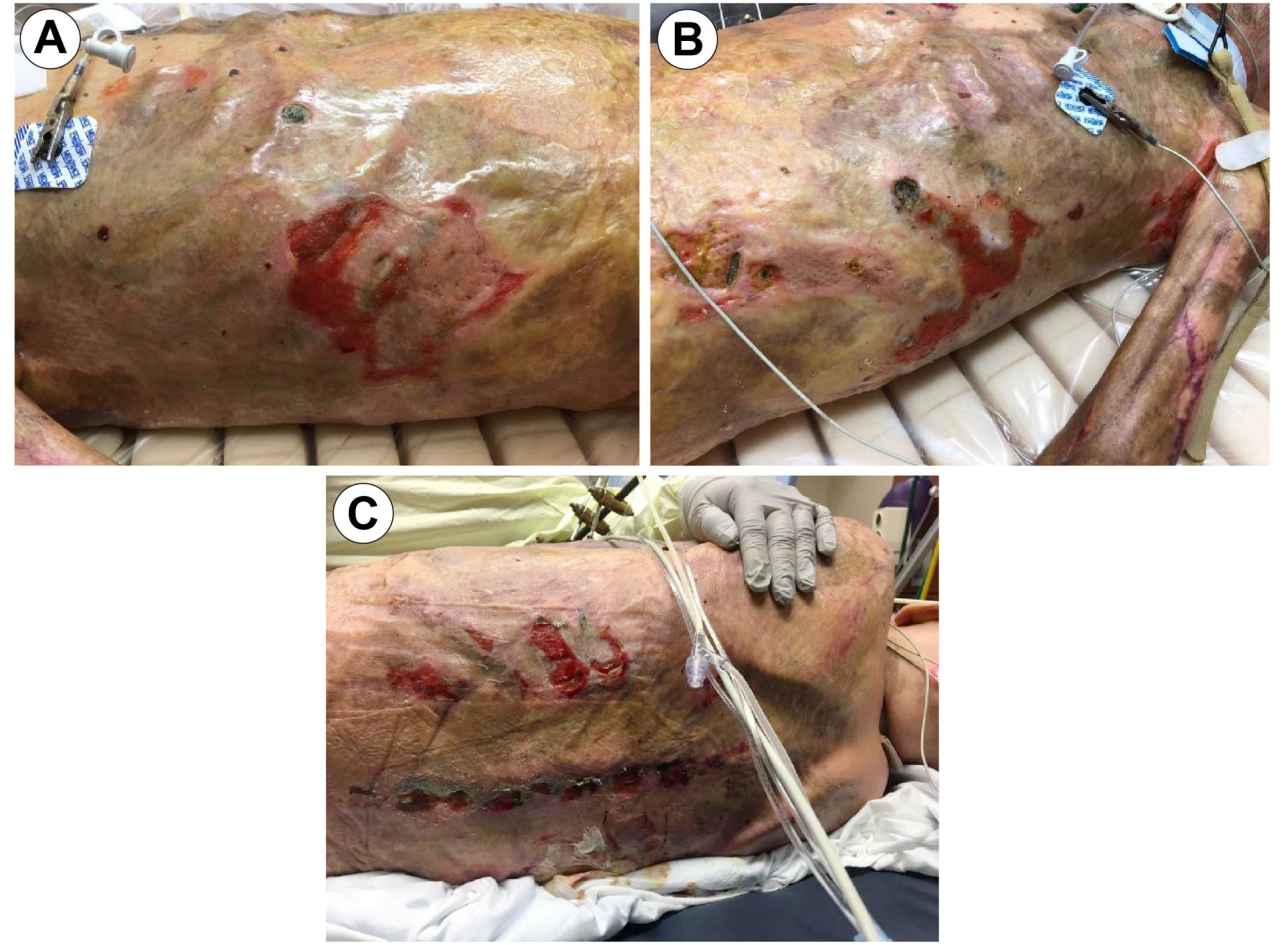
Six Months Post Burn Image A: View of Anterior Torso Right; Image B: View of Anterior Torso Left; Image C: View of Posterior Torso

Intravenous antibiotics were continued throughout the therapy trial, but topical antimicrobial coverage was stopped, which included Sulfamylon and silver nitrate soaks during the two-week period. In the following images, we see the patient at six weeks post-burn (Figure 8). Subsequently, we are able to graft and heal the posterior trunk and initiate physical therapy. The final image in this presentation is Figure 9, which was taken at six months post-burn.

## Discussion

Burn sepsis is the most common cause of morbidity and mortality in burn patients [6]. In patients with greater than 40% of the total body surface area (TBSA), 75% of all deaths can be attributed to sepsis from burn wound infection or other infection complications [9-10]. Both gram-positive and gram-negative species, Staphylococcus and Pseudomonas, are the most common bacteria observed in burn wound infections, as these species are commonly found in healthy skin flora. However, many other bacteria, fungi, and viruses can be a source of infection. The treatment of most burns includes a combination of debridement or excision, wound cleansing, and topical or systemic antimicrobial therapy. Since the introduction of early excision and the debridement of burn wounds, the incidence of sepsis has decreased [11].

Decreasing bacterial burden while safely treating the patient is the key to successful outcomes. Recent literature has shown that NPWT can be used as an adjunct to treat burn patients. NPWT can reduce the number of dressing changes for patients while improving management and healing time by stimulating the formation of healthy granulation tissue critical for successful grafting [12-13]. Current research has shown negative pressure wound therapy (NPWT) decreases the length of recovery time after burns, with a reported 78% decrease in hospital stay and a 76% decrease in cost [14]. Petkar and colleagues reported a higher mean graft take in NPWT patients compared to the conventional compression control group (96.7% vs. 87.5%; P <0.001) [15]. A study done by Chong and colleagues focused on the application of NPWT dressings for large areas of mixed burn wounds. The process named “total body wrap” reported wrapping limbs in large polyurethane dressing combined with NPWT. They showed that the wounds exhibited signs of re-epithelization in graft donor sites, decreased inflammatory exudate, and reduced exposure to pathogens by decreasing the number of dressing changes [16].

The introduction of NPWTi-d has given physicians a new tool to treat difficult wounds [12,17]. A study by Gabriel and colleagues employed the use of NPWTi-d therapy in patients with complex and infected wounds (>105 organisms) of the trunk or extremities. The results showed that wounds using NPWTi-d were able to clear infections faster (six days vs. 25.4 days; P <0.001) while subsequently reducing wound closure time (13.2 days vs. 29.6 days, P <0.001) [18]. In the assessment of NPWTi-d versus standard NPWT, Goss et. al were able to show a significant improvement in organism burden with NPWTi-d therapy when compared to standard NPWT therapy [19]. A paper published by Tutela and colleagues reviewed the use of antibiotic irrigation following breast reconstruction in 79 patients. Although looking at a different type of wound, the study reported a shorter time for primary wound closure after tissue damage. This team used a continuous 24-hour triple-antibiotic solution with an irrigation system, with irrigation or instillation times set for less than 30-minute intervals at a rate of 40 mL/hr [20].

The patient in this report presented with severe graft failure (95% loss) after surgical debridement and serious infection of the burn wounds. The authors use of NPWTi-d Veraflo therapy was beneficial to this patient by allowing immediate wound cleansing by removing slough, exudate, and infected material from the burn bed. After the two-week therapy had concluded and tissue cultures were negative for microbial growth, the wounds were grafted. The clinical outcome of this patient demonstrates the NPWTi-d Veraflo systems' efficacy in treating complex burn patients, with the instillation therapy playing a vital role in clearing the infectious bacteria from the wound.

The treatment of burn wounds is an evolving field, and new techniques, such as NPWTi-d, have shown great promise in the field of wound care. These therapies could prove to become a standard of care in complex burn patients presenting with serious infections after first-line treatments have been unsuccessful. This type of therapy is the new application of NPWTi-d, and a large-scale prospective study of NPWTi-d for burn patients is needed in order to prove its utility in a variety of burn wound presentations. A cost-benefit analysis would be useful to justify the increased use of NPWTi-d and to determine if the increased cost in acute management can decrease the cost of wound therapy in the long term. As new antimicrobial drugs and tissue engineering develop, NPWTi-d may be used as an ideal delivery system.

## Conclusions

NPWTi-d was a useful adjunct in treating and stimulating wound healing in a complex patient. This is the first case report of its kind utilizing a whole-body VAC with NPWTi-d, with successful results showing a decreased bacterial burden, decreased morbidity and mortality, and patient wound closure.

## References

[1] Branski LK, Herndon DN, Barrow RE (2012). A brief history of acute burn care management. Total Burn Care (Fourth Edition).

[2] World Health Organization (2011). Burn prevention. Success stories. Lessons learned. http://apps.who.int/iris/bitstream/handle/10665/97938/9789241501187_eng.pdf?sequence=1&isAllowed=y.

[3] Lionelli GT, Pickus EJ, Beckum OK, Decoursey RL, Korentager RA (2005). A three decade analysis of factors affecting burn mortality in the elderly. Burns.

[4] Rani M, Schwacha MG (2012). Aging and the pathogenic response to burn disease. Aging Dis.

[5] Wolf SE, Cancio LC, Pruitt B (2017). Epidemiological, demographic and outcome characteristics of burns [e-book]. Total Burn Care.

[6] Church D, Elsayed S, Reid O, Winston B, Lindsay R (2006). Burn wound infections. Clin Microbiol Rev.

[7] Alexander J, MacMillan B, Law E, Kittur D (1981). Treatment of severe burns with widely meshed skin autograft and meshed skin allograft overlay. J Trauma.

[8] McKanna M, Geraci J, Hall K (2016). Clinician panel recommendations for use of negative pressure wound therapy with instillation. Ostomy Wound Manage.

[9] Bang RL, Sharma PN, Sanyal SC, Al Najjadah I (2002). Septicaemia after burn injury: a comparative study. Burns.

[10] Gauglitz GG, Shahrokhi S, Williams FN (2017). Burn wound infection and sepsis. UpToDate.

[11] Sørensen B, Fisker NP, Steensen JP, Kalaja E (1984). Acute excision or exposure treatment?. Scand J Plast Reconstr Surg Hand Surg.

[12] Maurya S, Bhandari PS (2016). Negative pressure wound therapy in the management of combat wounds: a critical review. Adv Wound Care.

[13] Morykwas MJ, Argenta LC, Shelton-Brown EI, McGuirt W (1997). Vacuum-assisted closure: a new method for wound control and treatment: animal studies and basic foundation. Ann Plast Surg.

[14] Sabiston DC, Townsend CM (2016). Sabiston Textbook of Surgery: The Biological Basis of Modern Surgery. https://www.elsevier.com/books/sabiston-textbook-of-surgery/townsend/978-0-323-29987-9.

[15] Petkar KS, Dhanraj P, Kingsly PM (2011). A prospective randomized controlled trial comparing negative pressure dressing and conventional dressing methods on split-thickness skin grafts in burned patients. Burns.

[16] Chong SJ, Liang WH, Tan BK (2010). Use of multiple VAC devices in the management of extensive burns: the total body wrap concept. Burns.

[17] Kim PJ, Attinger CE, Olawoye O (2015). Negative pressure wound therapy with instillation: review of evidence and recommendations. Wounds.

[18] Gabriel A, Shores J, Heinrich C, Baqai W, Kalina S, Sogioka N, Gupta S (l2008). Negative pressure wound therapy with instillation: a pilot study describing a new method for treating infected wounds. Int Wound J.

[19] Goss S, Schwartz J, Facchin F, Avdagic E, Gendics C, Lantis II J (2012). Negative pressure wound therapy with instillation (NPWTi) better reduces post-debridement bioburden in chronically infected lower extremity wounds than NPWT alone. J Am Coll Clin Wound Spec.

[20] Tutela JP, Duncan DP, Kelishadi SS, Chowdhry S, Boyd T, Little JA (2015). Continuous postoperative antibiotic irrigation via catheter system following immediate breast reconstruction. Eplasty.

